# CYP3A5∗3 and C3435T MDR1 Polymorphisms in Prognostication of Drug-Resistant Epilepsy in Children and Adolescents

**DOI:** 10.1155/2013/526837

**Published:** 2013-08-01

**Authors:** Ewa Emich-Widera, Wirginia Likus, Beata Kazek, Paweł Niemiec, Anna Balcerzyk, Aleksander L. Sieroń, Iwona Żak

**Affiliations:** ^1^Department of Neuropediatrics, Medical University of Silesia, 16 Medykow Street, 40-752 Katowice, Poland; ^2^Department of Human Anatomy, Medical University of Silesia, 18 Medykow Street, 40-752 Katowice, Poland; ^3^Department of Biochemistry and Medical Genetics, School of Health Care, Medical University of Silesia, 18 Medykow Street, 40-752 Katowice, Poland; ^4^Department of General and Molecular Biology and Genetics, Medical University of Silesia, 18 Medykow Street, 40-752 Katowice, Poland; ^5^CoE Research and Teaching of Molecular Biology of Matrix and Nanotechnology, Network of CoE BioMedTech Silesia, 40-752 Katowice, Poland

## Abstract

Drug-resistant epilepsies still remain one of the most profound problems of contemporary epileptology. Several mechanisms of drug resistance are possible; among them, genetic factors have a prominent place. Much importance is attached to genes, which encode enzymes that metabolize antiepileptic drugs CYP 3A, which belong to the family of cytochromes P450 and the genome of multidrug resistance, such as multidrug resistance 1 (MDR1) that expresses P-glycoprotein (P-gp), a drug transporter protein. The aim of the study was to assess the relation between polymorphism of gene CYP3A5 and polymorphism C3435T of MDR1 gene with the occurrence of focal, drug-resistant epilepsy in children and youths up to 18 years of age. The study comprised 85 patients, and their age range was from 33 months to 18 years of age, suffering from epilepsy, partly responding well to treatment, partly drug resistant. The polymorphism of both genes has been analysed using the PCR-RFLP method. The study failed to corroborate association between polymorphism CYP3A5∗3 and C3435T polymorphism in MDR1 gene and pharmacoresistant epilepsy. The results of our research do not confirm the prognostic value of the polymorphisms examined in the prognostication of drug resistance in epilepsies.

## 1. Introduction

Epilepsy is one of the most frequently occurring diseases of the nervous system in children and youth [1–3]. Despite the enormous progress made in studies concerning epilepsy, and the introduction of new antiepileptic drugs (AEDs), it still remains impossible to achieve remission of epileptic fits in some patients, due to the problem of drug resistance [4, 5]. Neither clinical nor morphological factors can sufficiently explain this phenomenon. It seems that the genetic basis may not only predispose to the pathogenesis, but it also plays a role in the patient's response to AEDs [6–9]. The existence of a substantial heterogeneity of pharmacologic action of the same drug in specific persons can be explained with high probability by changes in patient's genotype, particularly when the enzyme that metabolizes the drug is encoded by a single gene, in the *locus* of which various allelomorphs may occur [10, 11]. 

Processes of oxidation of some 70% of the most often used drugs are catalyzed by one of microsomal enzymes—cytochrome P450. Cytochrome P450 enzymes primarily catalyze composed oxidation reactions, including some reductions and rearrangements of oxygenated species, for example, prostaglandins. The variability in drug metabolism, including antiepileptic drugs, is a result of the presence of many isoenzymes of cytochrome P450 family and their substantial polymorphisms [10–12]. Two enzymes CYP3A4 and CYP3A5 have the greatest importance in the metabolism of those drugs. They both participate in the metabolism of AEDs, such as: carbamazepine, oxcarbazepine, clonazepam, diazepam, phenobarbital, phenytoin, tiagabine, or zonisamide [13–15]. Clear personal variability (idiosyncrasy) in activity of CYP3A5 has been shown [16]. The polymorphism of CYP3A5 gene comprises several alleles. In overall population, the CYP3A5 alleles most frequently detected are the wild-type allele CYP3A5*1. A “mutant” allele CYP3A5*3 presents reduced enzymatic activity [10, 11]. Wild-type allele CYP3A5*1 encodes protein with full enzymatic activity [17]. The single base substitution in intron 3 at position 6986A>G resulting in different splicing leading to production of mRNA encoding a protein, with reduced enzymatic activity, or completely enzymatically inactive. The presence of mutant allele is the reason why the ability to drug metabolism is lost [18]. CYP3A5, is a dominating enzyme which constitutes about 30% of all P-450 cytochromes in liver and over 70% in the intestines. Polymorphisms and their link to drug resistance have not been investigated until now in epilepsies.

Lack of patient's response to AEDs may also be connected with genetically determined polymorphism of membrane proteins, participating in the transport of drugs through biological membranes, in particular those transporters classified to the family of ATP-binding cassette transporters also called ABC transporters, located within the intestine barrier area, as well as blood-brain barrier (BBB) area. In drug resistant epilepsy, importance is attributed to the polymorphism of (*multidrug resistance gene*) MDR1, encoding glycoprotein P [19–21]. The polymorphism in exon 26 C3435T is among the most often occurring ones, while its relation to drug resistance in epilepsies has been examined very intensely. It is a “silent” polymorphism, not leading to changes in the sequence of amino acids, yet connected with a change in P-gp expression and pharmacokinetics of drugs [22–24]. In patients with epilepsy, the MDR1 expression within the epileptogenic focus is significantly greater than in healthy tissue [19–22]. Numerous studies indicated the dependence between resistance to drugs and the occurrence of genotype CC or TT of the MDR1 gene, although at the same time the results of other analyses did not confirm that dependence [22–28].

The isoenzyme CYP3A5 is functionally and quantitatively related to CYP3A4, which has been connected with the expression of glycoprotein P and the resistance to AEDs; thus, it seems that also the polymorphism of CYP3A5 may have its share in the drug resistance mechanisms in epilepsy [15, 16, 29, 30]. CYP3A5, due to its common presence in peripheral and central compartments, is an important link in metabolism of AED. Probably, it is subject to common mechanisms, which regulate the transport of antiepileptic drugs, with the participation of MDR1; however, it has not been examined sufficiently, in particular in case of patients in developmental age. Taking into account the fact that the mechanism regulating the expression of CYP3A may be different for different races, for the purpose of studies concerning polymorphism and its connection with resistance to drugs, the authors have selected the CYP3A5*3 allele, which is an allele occurring most frequently in the Caucasian population [31]. 

 The aim of this study was to examine the connection between the polymorphism of CYP3A5 and C3435T MDR1 and the occurrence of resistance to drugs in children and adolescents with focal (partial) epilepsy. Additionally, the authors attempted to assess the cooccurrence of allele variants of gene CYP3A5 allele *1/*3 or *3/*3 and the CC, CT, or CT genotypes with resistance to drugs in epilepsy. 

## 2. Materials and Methods

### 2.1. Patients

 The studies on MDR1 polymorphism have been conducted on 85 patients with epilepsy, age range: from 33 months to 18 years (average age responders—11,4 ± 4,0 years, average age nonresponders—13,4 ± 4,5 years), of whom 38 were females and 47 males. Among those patients, 57 had epilepsy of unknown etiology, and 28 had symptomatic epilepsy. 

*The control group* consisted of a 100 healthy individuals, selected on the basis of their gender and age, with negative individual and family history, as regards epilepsy and febrile convulsions.

The polymorphism of gene CYP3A5 has been genotyped in 74 patients, age range: from 33 months to 18 years (average age responders—10,0 ± 4,3 years, average age nonresponders—8,4 ± 4,1 years) of whom 32 were females and 42 were males. Among those patients, 65 had epilepsy of unknown etiology, and 8 had symptomatic epilepsy.

*The control group* consisted of a 71 healthy individuals, selected on the basis of their gender and age, with negative individual and family history, as regards epilepsy and febrile convulsions.

All patients have administered carbamazepine (its concentration in blood serum has been determined) and/or oxcarbazepine. Moreover, the following have been also applied: valproic acid, vigabatrin, clobazam, lamotrigine, gabapentin, phenytoin, levetiracetam, phenobarbital, clonazepam, and topiramate. All drugs were administered at maximal doses, and they did not have unacceptable side effects. 

 When selecting patients, strict inclusion and exclusion criteria were followed. 

The inclusion criteria comprised: (1) sex: male and female, (2) diagnosis of partial epilepsy, in accordance with ILAE criteria (clinical manifestation arising no doubts as to the diagnosis of focal epilepsy and confirming EEG record), (3) known clinical response to the AED administered, (4) at least 2 years of patient's observation, (5) neuroimaging performed (at least MR). 

The exclusion criteria comprised (1) absence of unequivocal diagnosis of focal epilepsy, (2) idiopathic partial epilepsy, (3) progresse encephalopathy and (4) particular forms of the disease, such as: *epilepsia partialis continua*, particularly Rasmussen's syndrome.

 In this study, the authors applied the definition of resistance to drugs, proposed by the *International League Against Epilepsy*—ILAE [32]. Epilepsy in a child was assessed as drug resistant, if the therapy with the application of two drugs, tolerated well by the patient, properly selected for the patient and correctly administered, in monotherapy or polytherapy, failed and epileptic seizures continued. On the other hand, drug-sensitiveness was treated as freeing from seizures for the period of at least one year, or 3 times longer than the previous interparoxysmal period (whichever was longer).

### 2.2. Genotyping

 In isolated genomic DNA obtained from peripheral blood (Blood Mini, A&A Biotechnology), alleles CYP3A5*3 and polymorphism in C3435T in MDR1 gene were determined using the polymerase chain reaction-restriction fragment length polymorphism (PCR-RFLP).

### 2.3. Genotyping of CYP3A5

The CYP3A5*3 polymorphism was genotyped in accordance with the method described by Fukuen et al. [17], with modifications.

The genome DNA was amplified with the use of the following primers: CYP3A5 6956 Fm; 5′-CTT TTA AGA GCT CTT TTG TCT CTC A-3′ as *forward primer* and CYP3A5 7155 R; 5′-CCA GGA AGC CAG ACT TTG AT-3′ as *reverse primer*. The mixture, for reaction contained 25 *μ*L 10x PCR buffer, 02 mM dNTP's, 0.4 *μ*M of each primer, 100 ng of genomic DNA, and 1 unit of Taq DNA Polymerase (Fermentas). After denaturation at 94°C for 2 minutes, the amplification was carried out for 36 proper cycles at 94°C for 30 s, at 56°C for 15 s, and at 72°C for 30 s. The terminal elongation of DNA chains was carried out at 72°C for 5 minutes. After amplification of 5 *μ*L of the product (200 bp), it was annealed for 16 h at 37°C with the use of restrictive enzyme *DdeI* (Fermentas), obtaining fragments of 129, 107, 71, and 22 bp for allele *1, as well as 107, 71, and 22 bp for allele *3. The amplified fragments of DNA were separated in 8% polyacrylamide gel.

### 2.4. Genotyping of MDR1

The fragment of P-glycoprotein (197 bp) in exon 26 of MDR1 gene was amplified from genomic DNA with the sense primer: 5′-TGTTTTCAGCTGCTTGATGG-3′ and antisense: 5′-AAGGCATG TATGTTGGCCTC-3′. PCR amplification was performed in the total volume of 50 *μ*L containing 200 ng of genomic DNA, dNTPmix 10 mM/L, 5 pM/L each primers, 50 mM/L buffer with Mg^2+^, and polymerase DNA (DyNAzyme EXT PCR Kit, Finnzymes). The amplification reaction was performed using Mastercycler (Eppendorf AG, Hamburg, Germany). PCR amplification consisted of initial denaturation for 3 min at 94°C, followed by 34 cycles of denaturation at 94°C for 30 s, annealing at 60°C for 30 s, and extension at 72°C for 30 s. The terminal elongation was performed at 72°C for 7 min. After PCR amplification, the product (197 bp) was digested by Sau 3AI restriction enzyme (Fermentas) for 16 h at 37°C, generating fragments: 158 bp, 39 bp for allele 3435C; 197 bp, 158 bp, and 39 bp for allele C3435T. For the 3435T allele, we have not observed any digestion products. RFLP results were analyzed by electrophoresis, using 3.5% agarose gel [33].

### 2.5. Statistical Analysis

 The analysis was performed using SPSS Statistical software. The distribution of genotypes in patients and control group and their relationship with drug resistance were statistically evaluated by the *χ* ^2^ test with the Yate's correction. The genotype frequencies of the MDR1 polymorphism and CYP3A5 SNPs were tested for their fit to Hardy-Weinberg Equilibrium (HWE). A *P* value of < 0.05 was statistically significant.

## 3. Results 

**Figure 1:**
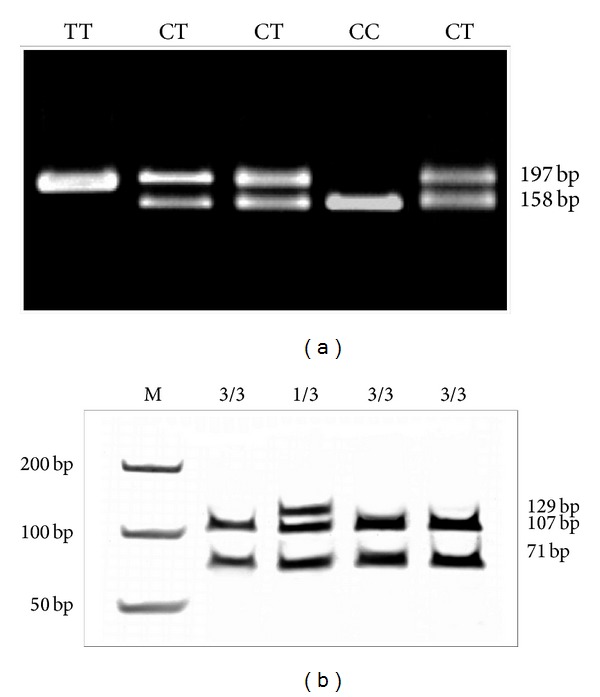
PCR-RFLP analyses for C3435T polymorphism MDR1 (a) and CYP3A5 variant alleles (b).

Analyzing the distribution of CYP3A5 alleles in the groups of patients with epilepsy, as well as in control group, we found statistically significant more frequent occurrences of homozygotes of CYP3A5*3/*3 than heterozygotes of CYP3A5*1/*3 (Table 1), (Figure 1(b)).

In both groups of patients, the presence of allele CYP3A5*1/*1 has not been noticed. Comparing the groups of patients who are drug resistant and who respond well to AEDs, no statistically significant differences have been found to exist between the frequency of occurrence of alleles *1/*3 and *3/*3 between the study groups (children with epilepsy) and control (*P* = 0.70). Comparing patients resistant to pharmacotherapy and those sensitive to drugs with the control group also revealed no statistically significant differences in both study groups (*P* = 0.38 and *P* = 0.98, resp.). Our analyses failed to confirm the connection between polymorphism of CYP3A5 with drug resistance in epilepsy. There were no statistically significant differences between sexes in CYP3A5 allele frequency presence. Analyzing the distribution of genotypes of C3435T polymorphism of the gene MDR1 in the population studied, the occurrence of three genotypes of the MDR1 gene has been found: CC, CT, and TT (Figure 1(a)). The distribution of genotypes in patients with epilepsy, as well as in the control group is presented in Table 2. 

The most frequently occurring genotype was the CT genotype, which, respectively, accounted for 55.0%—drug resistant patients, 64.0%—drug responsive patients and 51.0%—control. The CC genotype was the least frequent in both study groups, respectively: 15.0%—drug responsive patients, and 4.0% drug resistant patients. No statistically significant difference has been found between patients with epilepsy and the control group. There were no statistically significant differences between sexes in frequency of MDR1 gene alleles presence. 

Distributions of the frequency of occurrence of the examined alleles for MDR1 gene and CYP3A5 have been in compliance with Hardy-Weinberg equilibrium, both in control and in the study groups. 

 The cooccurrence of allele CYP3A5*1/*3 and CYP3A5*3/*3 has been analyzed for each C3435T genotype of the MDR1 gene: CC, CT, and TT. The statistical analysis confirmed lack of association between analyzed genotypes and resistance to AEDs in children and youths under 18 years of age (Table 3). 

## 4. Discussion

Among the enzymes responsible for the metabolism of antiepileptic drugs, an important role is played by the complex of cytochrome P-450. The activity of CYP3A is mainly influenced by two isoenzymes: CYP3A4 and CYP3A5, adjacent to each other on chromosome 7, yet their expression is independent [18]. The polymorphism of gene CYP3A5 often coexists with the polymorphic forms of gene CYP3A4. That connection is even stronger, as both isoenzymes take part in the metabolism of the same xenobiotics. Moreover, substantial activity of one isoenzyme may compensate for the reduced activity of another one [34]. CYP3A5 is characterized by a clearly individual variation of its activity and should be considered one of the elements of resistance to drugs [13, 34, 35]. The studies by Huang et al. [36] demonstrated that the ability to metabolize carbamazepine (CBZ) by CYP3A5 is comparable to that of CYP3A4. Park et al. [37], when conducting research in the group of patients, treated with CBZ, demonstrated that the presence of CYP3A5*1 allele results in the increase of CBZ concentration in blood serum (by 31% in comparison with others). The results indicate the influence of genotype of CYP3A5 upon the distribution of CBZ in patients with epilepsy. Similarly, Seo et al. [38], using the *population pharmacokinetic modeling,* demonstrated the influence of genotype of CYP3A5 upon the metabolism of CBZ in the Japanese population. Due to the fact that CBZ is an inductor of both expression and transcription of CYP3A4/5, the effect of CBZ activity is multidirectional. In the course of application of CBZ, initially, an increase of the concentration in proportion to the dose had been observed, and then, after the target dose was achieved, the concentration of the drug in blood serum diminished (inversely proportional to the dose applied), which may indicate a faster metabolism of the drug discussed due to the induction of CYP3A4/5 [37]. When looking for the influence of MDR1 upon the resistance to drugs, it is assumed that bioavailability of AEDs may be diminished as a consequence of excessive expression of proteins responsible for transport (e.g., MDR1) within the blood-brain barrier (BBB) [39, 40]. Most AEDs are such a weak substrate of P-gp, that, in principle, the constitutive expression of that protein on BBB is not able to reduce the inflow of AEDs in a clinically important way [41]; however, the inborn or acquired excessive expression P-gp in BBB may critically reduce the penetration of AEDs to the brain, resulting in resistance to many AEDs [41–43]. Such an over-expression of P-gp may result from the disease itself or the influence of AEDs of P-gp expression or the polymorphism of ABCB1. P-gp are encoded by the ABCB1 gene (MDR1), while its expression and function is connected with the polymorphism of ABCB1 C3435T; thus, SNP ABCB1 is the object of interest for pharmacogenetics. In addition, its excessive expression has been discovered on the surface of astrocytes and neurons of the brain tissue. The neuroglial localization of MDR transporters probably performs a protective function for cerebral cells [19, 44]. In patients with temporal lobe epilepsy, the overexpression of MDR1 has been found in hippocampus and the surrounding area. Long-lasting fits may induce excessive expression of P-gp and other transporter proteins in this location, which may explain the fact that patients are resistant to a wide spectrum of antiepileptic drugs, with various mechanisms of action, for example, to oxcarbazepine and phenytoin [45–47]. The results of the studies conducted so far, which analyze the connection between polymorphism of C3435T gene MDR1 with drug resistance in epilepsy, are contradictory. The meta-analysis conducted by Haerian et al. [26] comprising 22 studies on the connection between polymorphism of C3435T and resistance to drugs in epilepsy, conducted in the years 2003–2009, confirmed the relation between genotype CC and resistance to drugs. Hoffmeyer et al. [22] found that genotype CC caused excessive production of P-gp in the intestinal mucosa, while Siddiqui [48] demonstrated the same dependency within the blood-brain barrier. Kwan and Brodie [43] and Ufer et al. [49] demonstrated that genotype TT predisposes to the occurrence of resistance to drugs in epilepsies. The latest research, including meta analyses on large groups of patients with epilepsy—a group of 3000 patients—did not confirm the connection between the polymorphism studied and the resistance to antiepileptic drugs; however, that research concerned patients with various types of epilepsy, which may have influenced the results obtained [50]. The absence of connection between polymorphism of C3435T in the gene MDR1 in children was confirmed by the studies conducted by Chen et al. [51], Von Stülpnagel et al. [52], Vahab et al. [53], and Alpman et al. [54]. In accordance to Sánchez et al. [28], the resistance to AEDs depends on age and etiology of the epilepsy, yet that research failed to confirm the connection between the genotype studied and the resistance to drugs. The results of the research by Mohammed Ebid et al. [55] seemed promising, which demonstrated the dependency between the concentration of phenobarbital in PMR, depending on the genotype of ABCB1; for CC a significant reduction in drug concentration has been noticed, including the PMR/blood serum factor. The results of that study are in compliance with the study of Siddiqui [48] and confirm the assumption of connection between drug resistance and P-gp with a specific genotype. However, the latest study by Saygi et al. on groups of children with drug-resistant epilepsy and epilepsy reacting well to AEDs failed to confirm the connection between C3435T MDR1 polymorphism and the occurrence of resistance to drugs in children with epilepsy [56]. 

Studies of selected gene polymorphisms did not provide solid data for solving the problem of drug resistant pathomechanism for epilepsy. One mechanism under consideration is transporter, target and metabolic concept. The hypothesis on disturbed transport as causative factor for drug resistant epilepsy has been investigated mostly in the ABC-transporter. Still under consideration is non-ABC transporter, for example, RLIP76 and its influence on drug resistance issue [57]. 

The results of our research also do not support the connection of the studied polymorphism of gene MDR1 and resistance to drugs. In the group of children and youths under 18 years of age, none of the genotypes (CC, CT, and TT) appeared significantly more frequently in patients with drug-resistant epilepsy. No significant connection has been found to exist, neither in the entire examined population of children and adolescents with partial epilepsy, nor in the analysis comprising the additionally separated subgroups of patients with symptomatic epilepsy and epilepsy of unknown etiology. Our studies did not show a connection between the occurrence of the allele variant of CYP3A5 and resistance to drugs in partial epilepsy. 

## 5. Conclusion

 The research conducted does not support the significant prognosticating value of the studied polymorphisms of genes MDR1 and CYP3A5*3 in prognostication of pharmacoresistance of partial epilepsy in children. That area requires new hypotheses and new studies to be initiated. 

## Figures and Tables

**Table 1:** Isoforms of CYP 3A5 *3 versus drug resistance of epilepsy.

Isoforms of CYP	Number of patients		Control group [100%]
	Drug resistant [100%]	Drug responsive [100%]	
1.3.	10 (21.7%)	8 (28.6%)	22 (31.0%)
3.3.	36 (78.3%)	20 (71.4%)	49 (69.0%)
Comparison of groups of patients with epilepsy	NS (*P* = 0.70)		—
Comparisons with control group	NS (*P* = 0.38)	NS (*P* = 0.99)	—
Hardy-Weinberg equilibrium	Maintained (*P* = 0.15)	Maintained (*P* = 0.94)	—

NS: not statistically significant.

**Table 2:** Genotypes of C3435T MDR1 in study groups and drug resistance of epilepsy.

Genotype	Number of patients		Control group [100%]
	Drug resistant [100%]	Drug responsive [100%]	
CC	9 (15.0%)	1 (4.0%)	21 (21.0%)
CT	33 (55.0%)	16 (64.0%)	51 (51.0%)
TT	18 (30.0%)	8 (32.0%)	28 (28.0%)
Comparison of groups of patients with epilepsy (genotypes CC, CT, and TT versus other genotypes together)	NS CC (*P* = 0.28). CT (*P* = 0.51) TT (*P* = 0.612)		—
Comparisons with control group (genotypes CC, CT, and TT versus other genotypes together)	NS CC (*P* = 0.34) CT (*P* = 0.49) TT (*P* = 0.78)	NS CC (*P* = 0.10) CT (*P* = 0.24) TT (*P* = 0.74)	—
Hardy-Weinberg equilibrium	Maintained (*P* = 0.78)	Maintained (*P* = 0.33)	—

NS: not statistically significant.

**Table 3:** Cooccurrence of genotypes CYP3A5 and C3435T MDR1 and drug resistance in epilepsy.

Cooccurrence of CYP3A5 versus MDR1	Number of patients		Comparison group
	Drug resistant [100%]	Drug sensitive [100%]	
1.3 versus CC	0	0	NS
1.3 versus CT	17.7%	22.2%	
1.3 versus TT	5.9%	0	
3.3 versus CC	17.7%	0	
3.3 versus CT	52.8%	44.4%	
3.3 versus TT	5.9%	33.4%	

NS: not statistically significant.
